# Pointing Well

**Published:** 1863-06

**Authors:** 


POINTING WELL.—An article from the JOURNAL OF HEALTH has been going around all creation for nearly a year, commencing thus lucidly: "A person in good health, in the moderate pursuit of business, does not feel like drinking water, even in summer-time, if not very thirsty." We would like to know if any body ever felt like drinking water, summer or winter, if not thirsty. See what nonsense a dot and a letter can make a man talk. It should read thus: "Does not feel like drinking water even in summer, *is* not very thirsty." Make "if" "is," and omit comma after "water."

